# Predictive Ability of the Sexual Child Molestation Risk Assessment (SChiMRA+)

**DOI:** 10.1177/10790632261415813

**Published:** 2026-01-12

**Authors:** Allison McMahan, Timothy J. Luke, Gerhard Andersson, Christoffer Rahm, Malin Joleby

**Affiliations:** 1 Centre for Psychiatry Research, Department of Clinical Neuroscience, & Stockholm Health Care Services, Region Stockholm, 27106 Karolinska Institutet, Stockholm, Sweden; 2 Department of Psychology, University of Gothenburg, Gothenburg, Sweden; 3 Department of Behavioural Sciences and Learning, Department of Biomedical and Clinical Sciences, Linköping University, Linköping, Sweden

**Keywords:** child sexual abuse, psychometric properties, pedophilia, risk assessment, recidivism

## Abstract

In the field of child sexual abuse prevention, there is a need for risk assessment tools that are time-sensitive and can predict an individual’s risk of acting on sexual urges, whether this is watching child sexual abuse material (CSAM), interacting socially with a child for sexual purposes, or committing sexual abuse. We evaluated the predictive ability of the Sexual Child Molestation Risk Assessment (SChiMRA+), a self-report scale that assesses current motivation to act on sexual urges toward children (Part A) and captures past-week sexual behaviors toward children (Part B). Longitudinal data from two clinical trials aiming to reduce sexual behaviors toward children were analyzed; Priotab (testosterone-lowering medication; *n* = 52 men with pedophilic disorder; pseudonymized) and Prevent It (internet-delivered cognitive behavioral therapy; *n* = 160 active CSAM users; anonymous). A ROC analysis identified an optimal cutoff of 4.5 on Part A for indicating a clinically significant heightened risk for sexually abusive acts (same week: specificity 63.7%, sensitivity 83.7%, AUC = .802; next week: specificity 53.1%, sensitivity 83.0%, AUC = .727). These results indicate that SChiMRA+ part A can be used to identify increased risk for future offending behavior as well as probable ongoing CSAM use among self-identified help-seekers.

## Introduction

Digital developments, including encrypted networks, have reshaped the landscape and opportunity structure for committing sexual offenses, facilitating a substantial increase in the distribution of child sexual abuse material (CSAM). Consequently, it is crucial to advance our knowledge of the individuals perpetrating these crimes and identify appropriate risk assessment tools for the offense type. Research has historically focused on men convicted of contact offenses, but emerging studies highlight key differences between individuals committing contact offenses, individuals using CSAM, and those involved in both. Pedophilic interest remains a risk factor across all three groups (Babchishin et al., 2018; Hanson et al., 2016; Hanson & Morton-Bourgon, 2005; Ly et al., 2018; Seto, 2013), however there are noteworthy distinctions. Notably, the CSAM and mixed groups exhibit higher levels of pedophilic interest compared to the contact group (Babchishin et al., 2015; Seto, 2013). Another difference is that the CSAM offense group tends to present fewer antisocial tendencies and demonstrate greater victim empathy when compared to both the contact and mixed groups (Babchishin et al., 2015). When considering sociodemographic factors, the CSAM offense group is more likely to be single and without children (Ly et al., 2018) and have higher levels of education, employment, and income compared to the contact group (Ly et al., 2016). Research on the risk of individuals who have committed CSAM offenses also committing contact offenses is inconclusive. While some studies suggest that individuals with only CSAM offenses have a lower risk of reoffending compared to those with contact offenses or mixed offenses (Babchishin et al., 2015; Seto, 2013; Seto et al., 2011), concerns persist about potential escalation. In this context, a recent critical systematic review synthesizing 32 studies reported low prevalence of crossover among CSAM offenders (0.8–12%), higher rates among online-solicitation cohorts, and risk factors including pedophilic interest, prior criminal history, and greater severity and frequency of CSAM engagement (Ahmad et al., 2025). Consistent with a generally low crossover picture, some findings indicate a low crossover risk (Babchishin et al., 2018), whereas others, such as a recent darknet survey, report that a significant proportion of CSAM users seek direct online contact with children (Insoll et al., 2022). Finally, research suggests that individuals with CSAM-only offenses have relatively low rates of convictions for sexual recidivism after 5 years (3.9%), with reoffending more likely to involve new CSAM offenses (4.8%) rather than contact offenses (1.4%) (Helmus, 2023).

Accurately assessing risk is important for both clinical and judicial decision-making to protect any children at risk of being harmed. Over the last thirty years, multiple tools have been developed to predict the likelihood of sexual recidivism (Hanson & Morton-Bourgon, 2009; Hanson & Thornton, 2000; Kelley et al., 2020; Thornton et al., 2003). However, the majority of these assessments have been designed for a legal context and are based on statistics related to re-conviction rates. As a result, their applicability to non-convicted individuals is limited.

Of the currently available tools, the Static-99R, developed by R. Karl Hanson and David Thornton (2000), is one of the most researched, validated, and widely used risk assessment tools designed to predict the likelihood of sexual reoffending. The Static-99R was developed based on individuals who were charged, readmitted, or convicted between 1959 and the early 1990s and primarily focuses on verifiable facts such as static or historical factors, including age, prior criminal history, relationship status, and the nature of prior sexual offenses. The Static-99R has since been validated in different countries such as the US, Canada, Denmark, Singapore, and Australia (Helmus et al., 2022). However, it cannot be scored for individuals who have not been formally charged with contact or noncontact sexual offense directly involving a victim, including children (e.g. Eke et al., 2019). As such, it is thus not suitable for assessing those who have not yet committed an offence or only CSAM-related offenses. In addition, there is limited research supporting its use for predicting CSAM recidivism among individuals with a history of sexual crimes (Helmus et al., 2024).

Research on risk assessment for CSAM users is limited, with few validated tools available. While various tools are used across jurisdictions, most lack extensive validation or evidence. A noteworthy risk assessment tool that is specifically designed for CSAM related offenses is the Child Pornography Offender Risk Tool (CPORT). Developed by Eke et al. (2019), CPORT assesses the risk of recidivism among individuals convicted of CSAM-related offenses. Similar to Static-99R, CPORT relies on structured, verifiable factors, including prior offending history and offense characteristics, to provide an objective measure of risk. Unlike the Static-99R, which cannot be used for individuals without a formal charge for contact or noncontact sexual offenses involving a child, CPORT was developed to address this gap. CPORT is one of few assessments that have been evaluated in multiple studies (Brown, 2024). Helmus et al. (2024) identified eight tools that have predictive accuracy for CSAM recidivism, with CPORT remaining as one of the most validated, while also highlighting the overall limited evidence base currently in this field.

As these tools have been developed for and validated primarily in convicted populations, they may be unsuitable for risk assessment in cases where archival data are unavailable. For example, many currently used tools are not tailored for assessing the risk in self-identified, help-seeking patients without knowing whether they have a prior conviction history, or to monitor changes in risk over short periods, for example, during the course of treatment or participation in a clinical trial. Assessing risk in a clinical context is crucial as it ensures that treatment is matched with an individual’s risk of reoffending, following a model that considers both their risks and their needs (Bonta & Andrews, 2017) and that appropriate resources are allocated to protect children at risk of harm.

One way of assessing risk without accessing archival data is through self-report. However, this is often met with skepticism in forensic contexts due to concerns about accuracy, as individuals may alter their responses due to social desirability biases, fear of legal consequences, or other types of reluctance to disclose sensitive information (Tourangeau & Yan, 2007). This can impact the reliability of self-reports, particularly when assessing criminal behaviors or risk factors associated with recidivism. At the same time, motivation and self-regulation are recognized as dynamic and clinically relevant factors for recidivism risk and treatment engagement in forensic risk assessment (Ginsburg et al., 2002; Thornton, 2013), yet they are difficult to capture with archival sources. Consistent with this, empirical evidence suggests that, when used in a structured manner alongside other assessment methods, self-report measures retain value in forensic, correctional, and psychiatric settings (Kroner et al., 2020; Rodrigues et al., 2016; Walters, 2006). While research has shown that risk assessments that incorporate historical and non-self-report data tend to be more predictive of recidivism, well-designed content-relevant self-report instruments remain useful in providing meaningful insights into criminal thinking patterns, aggression, attitudes, and antisocial behaviors (Walters, 2006). Additionally, structured self-report tools have been found to facilitate transparency and client engagement in the assessment process, helping to improve the accuracy of risk assessments and intervention planning (Kroner et al., 2020). There is also evidence supporting the predictive validity of self-report measures in both institutional settings and post-release contexts, with findings indicating their ability to assess risks related to institutional misconduct and general recidivism (Rodrigues et al., 2016). Evidence from samples of men convicted of sexual offences indicates that confidential self-report can provide risk-relevant information that broadly agrees with official records and can be used to approximate scoring on established risk tools (Pham et al., 2020). This supports cautious, structured use of self-report alongside other sources when file information is limited. Therefore, while there are limitations to self-report assessments, they still can be expected to provide valuable information for evaluating risk.

### Addressing Gaps With SChiMRA+

To address current gaps in risk assessment, the *Sexual Child Molestation Risk Assessment* (SChiMRA) was initially developed by one of the authors (CR) as a pen-and-paper tool (Landgren et al., 2020) and was later updated and adapted to the online version, SChiMRA+. These assessments allow for time-sensitive self-reported risk assessment without relying on archival data, such as prior convictions (Wittström et al., 2020). SChiMRA + has been shown to be suitable for clinical trials as it demonstrates sensitivity to change during short time intervals (weeks). For example, expected change is seen in both testosterone lowering medication (Landgren et al., 2020) and internet-based cognitive behavioral therapy (Lätth et al., 2022), as well as, to a lower degree, in control conditions such as pharmacological placebo (Landgren et al., 2020) and psychological placebo (Lätth et al., 2022).

The SChiMRA and SChiMRA + are self-report questionnaires designed for help-seeking individuals that focuses on self-reported motivation to engage in sexual urges (Part A) and/or self-reported sexual behaviors towards children (Part B), including fantasizing about children, use of CSAM, socializing with children, or sexual contact with children. These two parts are meant to demonstrate ‘motivation’ and ‘acting’ as neurobiological concepts, drawing from the Incentive-Sensitization Theory of Addiction (Berridge & Robinson, 2016). In this theory, ‘acting’ or ‘liking’, refers to the individual’s consummatory behavior, while ‘motivation’ or ‘wanting’ pertains to the motivational aspects driving that behavior. These components aim to capture the nuanced nature of sexual urges towards children. Complementing this neurobiological theory, psychological theories emphasize motivation as a proximal but distinct driver of behavior. Within the Motivation-Facilitation Model of sexual offending, primary sexual motivations (e.g., paraphilias, high sex drive, intense mating effort) energize behavior, while trait and state facilitators (e.g., antisociality, self-regulation problems, intoxication, negative affect) and situational opportunity determine whether that motivation is acted upon (Seto, 2019).

Consistent with this distinction, Part A of SChiMRA+, which assesses self-reported motivation, operationalizes a proximal motivational state under minimal-inhibition assumptions *(“how likely are you to… if there were no negative consequences)*. This framing intentionally decouples desire from immediate legal concerns to approximate the motivational drive that, according to the Motivation-Facilitation Model, precedes action. In contrast, Part B captures enacted behavior (*how many times during the past seven days have you engaged in any of the following…?*). This dual structure approach is also consistent with the Theory of Planned Behavior, in which motivation, together with attitudes, subjective norms, and perceived behavioral control, predicts intentions and action (Ajzen, 1991), as well as with Self-Determination Theory, which posits that intrinsic and extrinsic motivation shape goal-directed behavior (Deci & Ryan, 2000). Together, these psychological, neurobiological and forensic perspectives support the inclusion of motivation and behavior as independent constructs in SChiMRA+ and their respective roles in short-interval monitoring of sexual urges and sexual behaviors toward children.

### The Present Study

This study aims to evaluate the predictive validity of SChiMRA + for both CSAM-related and other sexual offending behaviors against children, using data from two previous clinical trials: Priotab and Prevent It. Specifically, we hypothesize that: Higher scores on SChiMRA + Part A (self-rated motivation) will predict increased engagement in problematic behaviors as captured in SChiMRA + Part B (self-reported behaviors). By examining this hypothesis, we aim to assess whether SChiMRA + can improve risk assessment for non-convicted individuals and contribute to the broader effort to prevent child sexual abuse.

## Methods

### Ethical Considerations

All research in both Priotab (no. Ö26-2014) and Prevent It (no. Ö2019-1) was approved by the Swedish Ethics Review Appeals Board. Adhering to Swedish law, the research team followed mandatory reporting obligations, ensuring that any disclosed instances of abusive behaviors were addressed in accordance with legal requirements. In cases where a criminal act was reported alongside identifiable information that could potentially warrant reporting, the university’s legal team, in consultation with the Principal Investigator, made informed decisions on whether or not to report, emphasizing a careful and ethical approach to handling sensitive information.

### Procedure

In order to test the SChiMRA and SChiMRA + self-report’s predictive ability, we used data from two previous clinical trials: Priotab and Prevent It. Priotab was a medical intervention study conducted to determine whether a gonadotropin-releasing hormone antagonist could be effective in reducing dynamic risk factors for committing CSA (Landgren et al., 2020). Participants were recruited at the ANOVA center at Karolinska University Hospital, Stockholm, Sweden, through their national telephone helpline, PrevenTell. This clinical trial was a double-blind, placebo-controlled, parallel-group phase 2 trial with balanced randomization, which compared a group receiving injections of 2*120 mg of degarelix acetate to those receiving an equal volume of placebo, and followed for 10 weeks during the years 2016-2019. Pseudonymization was done so that the research nurse checked the ID document at each visit to ensure that it was the same research individual who participated each time. In the medical record system, however, the individual was assigned an anonymous study ID, and their identity was not disclosed to others, including researchers who interacted with the participants. These participants were help-seeking, self-identified men with a pedophilic disorder diagnosis. Participants answered questionnaires at baseline, 2 weeks, and 10 weeks.

Prevent It was an eight-week, internet-based cognitive behavioral therapy (CBT) program for mainly non-convicted and anonymous adult CSAM users who wanted help to stop CSAM use. Participants were recruited from encrypted onion sites on the darknet, utilizing the onion services protocol on the Tor browser to ensure enhanced anonymity. The therapy aimed to reduce the use of CSAM by enhancing the management of sexual urges and risk situations, strengthening coping strategies, and focusing on living a value-directed life. Each week, the patients received new module content, assignments, a battery of assessments (including SChiMRA+) and individual therapist feedback on the previous module. Participants answered questionnaires at baseline, during 7 weekly assessments, post-treatment, and at a 4-week follow-up. This randomized placebo-controlled trial showed Prevent It to be effective in decreasing the consumption of CSAM and well appreciated by the patients (Lätth et al., 2022). Patients were also asked to rate their truthfulness in how they had answered the assessments (0–10, with 0: not truthful at all; 10: 100 % truthful), and results indicated a high level of self-reported truthfulness across the groups when measured at pre-treatment (Prevent It: *N* = 73, M = 9.5, SD = 1.2; Placebo: *N* = 78, M = 9.3, SD = 0.97), post-treatment (Prevent It: *N* = 35, M = 9.6, SD 0.55; Placebo: *N* = 51, M = 9.5, SD = 0.86), and follow-up (Prevent It: *N* = 16, M = 8.8, SD 1.5; Placebo: *N* = 23, M = 9.3, SD = 0.83).

Using the data sets from both clinical trials, we evaluated the ability of the SChiMRA + motivation items at the time of reporting to predict self-reported behavior such as CSAM use, socializing with a child for sexual arousal or in the hopes it may later lead to something more, or interacting sexually with a child in the week prior to reporting. The week after reporting was also analyzed for the Prevent It data set, where there was weekly information available. The determination of sample size, rules for stopping data collection, and criteria for case exclusion were established by the two original trials, and our analyses were not pre-specified prior to data collection. We take responsibility for the integrity of the data, the accuracy of the data analyses, and have made every effort to avoid inflating statistically significant results. We have established a Supplemental Materials page on open science framework (https://osf.io/aydmu/). Here we have included the R code used for all analyses, as well as additional analyses where we have examined potential moderating effects of the treatments and found no evidence that the reported associations vary as a function of treatment.

### Participants

Priotab included 52 pseudonymized male participants (aged 18–66) from Sweden with diagnosed pedophilic disorder. Prevent It recruited globally and included 160 anonymous predominantly male (98%) participants (aged 18–59), from Europe (47%), North America (33%), Asia (9%), Oceania (4%), South America (4%) and Africa (1%), that were viewing CSAM within the week prior to the interview. See Table 1 for patient demographics.

**Table 1.** Patient Demographics

	Priotab *N* (%)	Prevent it *N* (%)
Number of participants	52	160
Age	15 (28) were 18–29	78 (49) were 18–29
	24 (44) were 30–39	47 (30) were 30–39
	8 (15) were 40–49	23 (15) were 40–49
	4 (7) were 50–59	10 (6) were 50–59
	3 (6) were 60–69	0 (0) were 60–69
Gender		
Male	52 (100)	157 (98)
Non-binary	-	2 (1)
No reported gender	-	1 (.5)
Psychiatric morbidity		
Ongoing substance misuse^a^	15 (29)	46 (29)
Ongoing major depression^b^	19 (37)	53 (33)
Any other current psychiatric comorbidity	43 (83)	107 (67)
Sexual behavior and interest		
Pedophilic interest^c^	52 (100)	129 (81)
Pedophilic disorder^d^	52 (100)	91 (57)^e^
Self reported prior convictions		
Contact sexual offense	5 (10)	4 (3)
Non-contact sexual offense	8 (15)	7 (4)
Non-sexual offense	7 (13)	6 (4)
Recruitment method	PrevenTell helpline	Mostly darknet forums related to CSA and CSAM
Treatment	Degarelix: *n* = 25 (48), with 1 withdrawal; placebo: *n* = 26 (50)	Prevent it CBT: *n* = 76 (48); placebo: *n* = 78 (49)

Data format: *N* (%).

^a^Measured by The Alcohol Use Disorders Identification Test (AUDIT) and The Drug Use Disorders Identification Test (DUDIT)

^b^Measured by the Montgomery-Åsberg Depression Rating Scale (MADRS) in Priotab and Depression sub-questionnaire in The Mini International Neuropsychiatric Interview (MINI) in Prevent It.

^c^Indicated agreement by responding somewhat or completely to the statement “I have had sexually arousing fantasies about, or urges to, have sex with someone who is prepubescent and at least 5 years younger than myself” within a self-report questionnaire addressing various paraphilic sexual interests, as per DSM-IV/5 diagnostic criteria.

^d^Diagnosed pedophilic disorder was an inclusion criteria for Priotab. No patients in Prevent It were formally diagnosed.

^e^Probably pedophilic disorder in Prevent It, deemed to fulfill DSM-IV/5 diagnostic criteria based on the self-report questionnaire and pre-treatment data by: (i) meeting inclusion criteria of being 18 years or older and engaging in CSAM use within the past week, (ii) satisfying the criterion for pedophilic interest outlined above or responding affirmatively to the statement “I have had sex with someone who is prepubescent (and at least 5 years younger than myself),” and (iii) indicating agreement to the statement “At some point this lasted for a period of six months or more.”

### Measures

#### Sexual Child Molestation Risk Assessment (SChiMRA+)

The *Sexual Child Molestation Risk Assessment* (SChiMRA+) is a questionnaire for clinicians to have a time sensitive measure of a patient’s self-rated sexual urges and behaviors during treatment and that captures hard outcome measures (i.e. actual offending behavior). SChiMRA+ is the second version of the SChiMRA which expands on different items and allows for delivery online, these differences are detailed below, and both versions of the assessment can be found in the supplementary. SChiMRA+ was developed for anonymous help seeking individuals and is available in English, Swedish, German, Finnish, Portuguese, Spanish, Czech, and Slovak. This measure has not yet been used in reliability or validity trials. Part A of the questionnaire measures self-rated risk at the time of assessment and is related to ‘motivation’ to engage with children (e.g. “How likely is it that you would do any of the following if there was an easy way to do it without being detected?”). Part B measures self-reported behavior in the week prior to assessment, and covers past week behaviors, or ‘acting’ (e.g. “Think about the last seven days. How often have you engaged in some of the following behaviors”, “How much time was spent during each day?”), including time spent using child sexual abuse material (CSAM), socializing or physically interacting with children for sexual arousal. Part B in SChiMRA + also includes a self-rated version of the Combating Paedophile Information Networks in Europe (COPINE) scale (Quayle, 2008) which measures the severity of CSAM viewed from 1–10 (1 = Indicative, 2 = Nudist, 3 = Erotic, 4 = Posing, 5 = Erotic posing, 6 = Explicit Erotic Posing, 7 = Explicit Sexual Activity, 8 = Assault, 9 = Gross Assault, 10 = Sadistic/Bestiality), as well as an estimation of the age of the youngest child in the CSAM viewed that week (measured in years), and related behaviors (such as searching for CSAM, interacting/discussing with other people about one’s sexual interest in children, categorizing CSAM or fantasizing about children).

#### Static-99R

The Static-99R is a widely used risk assessment tool designed to estimate the likelihood of sexual recidivism among adult males convicted of sexual offenses. It consists of ten items that assess various static (historical, unchangeable) risk factors, such as age at release, prior sexual offenses, and relationship history. The Static-99R has been extensively validated for use in predicting contact sexual offenses among convicted individuals (Helmus et al., 2022). However, its applicability to individuals who have engaged in CSAM-related behaviors is limited. Research suggests that the Static-99R does not effectively assess the risk of CSAM offending, as it was developed based on contact sexual offense populations and does not include dynamic or offense-specific factors relevant to CSAM use (Helmus et al., 2024). Due to these limitations, caution is advised when interpreting its scores in populations that primarily engage in online sexual offenses, such as CSAM users.

#### Implementation of SChiMRA and Static-99R* in Priotab

In the Priotab clinical trial, the first version of the SChiMRA was used. In this first version of SChiMRA, a pen-and-paper assessment, self-reported risk was measured on a visual-analogue scale (0–114 mm) when patients responded to the question, “How likely is it that you would do any of the following, if there was an easy way to do it without being caught? Mark an X on the line.” The risk was interpreted as clinically significant if rated at or above 40% (i.e. >45 mm). This method was inspired by Serlin et al. (1995) where the standard operational definition for ‘significant pain’ was greater than 4 of 10 on a VAS, the point patients describe substantially more interference with their function. It covered the three domains of watching CSAM or observing children with sexual intentions; socializing with children with sexual intentions; and direct sexual interaction with children. Part B was self-rated on a 4-point Likert scale, with the response options 0 (=never), 1 (=several days), 2 (=more than half of days), and 3 (=almost every day) on the same domains. The total score ranges from 0 to 9.

The Static-99R assessment was used in the Priotab clinical trial, but without having access to criminal records. The questions were administered and scored in-person during the initial screening by a medical professional (see Landgren et al., 2020). Because it was applied to a non-validated population, and without access to records, we will refer to its use as Static-99R*.

#### Implementation of SChiMRA+ and Static-99R* in Prevent It

In the Prevent It clinical trial the second version of the SChiMRA, SChiMRA+, was used. The differences include having a 10 point scale (0 being not at all to 10 being very likely) instead of a visual scale, self-rating on a continuous scale asking for the number of days and hours acting on different behaviors instead of a 4-point Likert scale, the inclusion of rating the material according to the COPINE scale, and lastly that the SChiMRA+ is given online instead of on paper. This expansion allows for more information for the evaluator or therapist, but the scoring system remains the same.

The Static-99R questions used in the Prevent It clinical trial were incomplete, and there was no access to criminal records of the participants. The questions were administered and scored during the initial intake interview, by one of the therapists involved in the clinical trial (see Lätth et al., 2022). Questions 8, 9, and 10 (whether victims were unrelated, strangers, or male), were not asked in the intake interview, which could affect the predictive ability. Because it was applied to a non-validated population, and without access to records, we will refer to its use as Static-99R*.

### Statistical Analyses

#### Priotab

Using all valid data across 3 measurement points (at baseline, 2 weeks and 10 weeks), we fit linear mixed effects models predicting self-reports from the SChiMRA Part B: (1) the use of CSAM over the last week (*n* = 156 observations from *N* = 55 participants), (2) socialization with children over the last week (*n* = 156 observations from *N* = 55 participants), and (3) sexually interacting with children over the last week (*n* = 156 observations from *N* = 55 participants). Each of these outcomes were predicted by the ratings from the SChiMRA Part A. Each model featured random intercepts for each participant. Given the limited amount of data, we did not add random slopes to the models, to minimize model complexity. Mixed effects models were fit using the lme4 package (Bates et al., 2015) for R (R Core Team, 2023), supplemented with the lmerTest package (Kuznetsova & Christensen, 2017) to calculate Satterthwaite degrees of freedom for significance tests of the coefficients. Mixed effects models allow for the use of data from all measurement points in the study, while accounting for non-independence of observations. This feature is useful for increasing statistical power, and where sufficient data are available, these models also allow us to examine the extent to which effects vary across persons.

Additionally, we fit linear mixed effects models to examine the extent to which total scores from the Static-99R* predicted CSAM use, socializing with children, and interacting sexually with children on Part B of the SChiMRA. These models used all valid data across the 3 measurement points, and featured random intercepts for each participant.

#### Prevent It

To assess the extent to which self-reported motivation predicted behavior, using all valid data from the 9 weekly measurement points across the trial (at baseline, 7 weekly assessments, and post assessment), we fit linear mixed effects models predicting self-reports from the SChiMRA + Part B: (1) the number of hours of CSAM watched over the last week (*n* = 894 observations from *N* = 154 participants), (2) the average COPINE rating of the CSAM watched over the last week (*n* = 650 observations from *N* = 152 participants), and (3) the average estimate of the age of the youngest child (measured in years) featured in the CSAM watched over the last week (*n* = 653 observations from *N* = 152 participants). Each of these outcomes was predicted by the three grand mean centered ratings from the SChiMRA + Part A from the same measurement point, assessing the motivation to watch CSAM, to socialize with children with the intent to have later sexual interactions, and to sexually interact with children. Additionally, each model featured random intercepts for each participant and random slopes for each of the three predictors for each participant.

Additionally, to examine the extent to which self-reported motivation predicted future behavior, we fit linear mixed effects models predicting the behavior estimates from the SChiMRA + Part B for the next week (CSAM use, *n* = 651 observations from *N* = 126 participants; average COPINE rating, *n* = 441 observations from *N* = 116 participants; average youngest child, *n* = 444 observations from *N* = 116 participants). Because these models involved fewer valid observations, the models were simpler than those using data from the same measurement point. Each outcome was predicted by self-reported motivation to watch CSAM, with random intercepts for each participant and random slopes for motivation to watch CSAM for each participant.

Similar to the approach taken with the data from the Priotab trial, we fit linear mixed effects models to examine the extent to which the measure of static risk based on the Static-99R* predicted CSAM use, average COPINE ratings, the average youngest child reported, socializing with children, and interacting sexually with children on Part B of the SChiMRA+. These models used all valid data across the 9 measurement points and featured random intercepts for each participant and random slopes for Static-99R* for each participant.

We also performed a receiver operating characteristic (ROC) analysis to find optimal cutoffs for using self-reported motivation to watch CSAM to predict whether a participant used CSAM at the same measurement point and prospectively (i.e., in the next week). Participants were coded as having used CSAM if they reported any non-zero use of CSAM on Part B of the SChiMRA+. To establish the cutoff values, we used Youden’s (1950) method. ROC analyses were performed using the *pROC* package (Robin et al., 2011) for R.

## Results

### Priotab

#### SChiMRA Part A (“Wanting”) Predicting SChiMRA Part B (“Acting”)

The results of the analyses of the SChiMRA Part A items predicting the Part B items within the same measurement point are reported in Table 2. As can be seen in the table, motivation to watch CSAM predicted self-reported use of CSAM. Motivation to socialize with children predicted self-reported socializing with children. Motivation to interact sexually with children predicted self-reported sexual interactions with children. Motivation to interact sexually with children also appeared to negatively predict socializing with children, however, the bivariate relationship between these variables was small yet positive, *r* = .08. The negative sign of this regression coefficient is due to suppression from the inclusion of motivation to socialize in the model. We did not conduct a ROC analysis with the Priotab data because there were insufficient observations in the Priotab data.

**Table 2.** SChiMRA Part a “Wanting” Predicting SChiMRA Part B “Acting” Within the Same Week in the Priotab Clinical Trial: Results of Linear Mixed-Effects Models

Behavior: Weekly CSAM use			
Term	Estimate [95% CI]	t (df)	*p*
*Fixed Effects*	*b*		
Intercept	0.01 [−0.20, 0.21]		
Motivation to watch	0.09 [0.06, 0.13]	5.68 (151.70)	< .001
Motivation to socialize	0 [−0.07, 0.07]	0.09 (135.98)	.929
Motivation to interact	−0.02 [−0.09, 0.05]	0.57 (140.90)	.572
*Random Effects*	*SD*		
Participant intercepts	.49		
Residual	.47		

Behavior: Socializing with children			
Term	Estimate [95% CI]	t (df)	*p*
*Fixed Effects*	*b*		
Intercept	−0.03 [−0.10, 0.04]		
Motivation to watch	0.01 [0.00, 0.02]	1.58 (119.61)	.116
Motivation to socialize	0.09 [0.06, 0.12]	6.07 (151.94)	< .001
Motivation to interact	−0.07 [−0.10, −0.04]	4.89 (150.84)	< .001
*Random effects*	*SD*		
Participant intercepts	.11		
Residual	.23		

Behavior: Sexually interacting with children			
Term	Estimate [95% CI]	t (df)	*p*
*Fixed effects*	*b*		
Intercept	0.00 [−0.02, 0.02]		
Motivation to watch	0.00 [0.00, 0.00]	.063 (101.83)	.950
Motivation to socialize	−0.02 [−0.03, −0.01]	4.16 (144.72)	< .001
Motivation to interact	0.02 [0.01, 0.03]	4.68 (140.57)	< .001
*Random effects*	*SD*		
Participant intercepts	.01		
Residual	.07		

*Note.* For fixed effects, estimates are unstandardized regression coefficients. For random effects, the standard deviation of the intercepts is displayed. Behavior was measured on a 0 to 3 scale.

#### Static Risk

The Static-99R* used in the Priotab trial did not significantly predict CSAM use, *b* = 0.02 [−0.07, 0.11], *t* (54.93) = 0.44, *p* = .663, socializing with children, *b* = 0.02 [−0.01, 0.04], *t* (53.32) = 1.23, *p* = .223, or sexually interacting with children, *b* = 0.00 [−0.01, 0.01], *t* (52.30) = 0.31, *p* = .761.

### Prevent It

#### SChiMRA + Part A (“Wanting”) Predicting SChiMRA + Part B (“Acting”)

**Figure 1.**
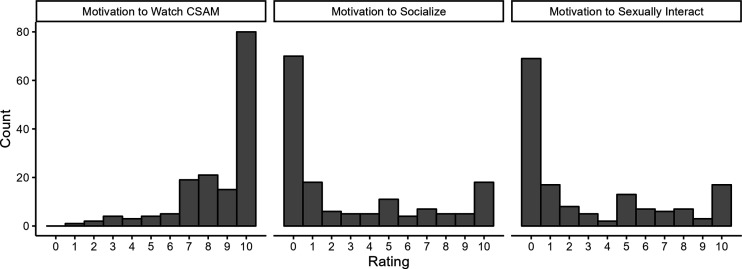
Frequency of prevent it treatment and placebo patients self-rated motivation to watch CSAM from 0-10 at baseline *Note. N* = 154

Table 3 displays the results of the linear mixed effects models predicting behaviors with the “wanting” items on the SChiMRA + Part A from self-reports taken at the same weekly measurement point as well as at the next week. As can be seen in Table 3, motivation to watch CSAM as well as motivation to interact with children predicted the number of hours of CSAM watched measured at the same time point, and motivation to watch CSAM predicted use of CSAM in the following week. Motivation to watch CSAM also predicted average COPINE severity (at the same time point) and the average age of the youngest child in CSAM (both at the same time point and in the following week). However, none of the SChiMRA + Part A items significantly predicted socializing with children or sexually interacting with children. Given that these behaviors were relatively rare in this sample, this result may be due in part to a lack of variance. Figure 1 shows the number of participants in the treatment and placebo conditions who had motivation to watch CSAM, socialize, or sexually interact with children at the baseline measurement.

**Table 3.** SChiMRA Part A “Wanting” Predicting SChiMRA Part B “Acting” Within the Same Week and in the Following Week in the Prevent It Clinical Trial: Results of Linear Mixed-Effects Models

Predicting at same time point				Predicting next week			
Behavior: Total hours of CSAM							
Term	Estimate [95% CI]	t (df)	*p*	Term	Estimate [95% CI]	t (df)	*p*
*Fixed effect*	*b*			*Fixed effects*	*b*		
Intercept	3.06 [2.51, 3.61]			Intercept	1.13 [0.34, 1.92]		
Motivation to watch	0.40 [0.30, 0.50]	8.02 (177.71)	< .001	Motivation to watch	0.20 [0.10, 0.30]	3.93 (624.62)	< .001
Motivation to socialize	0.06 [−0.1, 0.22]	0.73 (185.65)	.464				
Motivation to interact	0.37 [0.14, 0.6]	3.23 (118.01)	.002				
*Random effects*	*SD*			*Random effects*	*SD*		
Participant				Participant			
Intercept	3.05			Intercept	2.19		
Motivation to watch	0.38			Motivation to watch	.10		
Motivation to socialize	0.16			Residual	2.84		
Motivation to interact	0.63						
Residual	2.52						

Behavior: Weekly average COPINE severity							
Term	Estimate [95% CI]	t (df)	*p*	Term	Estimate [95% CI]	t (df)	*p*
*Fixed effect*	*b*			*Fixed effect*	*b*		
Intercept	6.98 [6.61, 7.35]			Intercept	6.84 [6.42, 7.27]		
Motivation to watch	0.20 [0.11, 0.28]	4.70 (70.25)	< .001	Motivation to watch	0.07 [0.00, 0.15]	1.89 (46.83)	.065
Motivation to socialize	−0.01 [−0.1, 0.08]	0.28 (97.50)	.782				
Motivation to interact	0.06 [−0.03, 0.14]	1.26 (202.87)	.208				
*Random effects*	*SD*			*Random effects*	*SD*		
Participant				Participant			
Intercept	2.02			Intercept	2.10		
Motivation to watch	0.22			Motivation to watch	0.05		
Motivation to socialize	0.07			Residual	1.37		
Motivation to interact	0.01						
Residual	1.28						

Behavior: Weekly average youngest child in CSAM							
Term	Estimate [95% CI]	t (df)	*p*	Term	Estimate [95% CI]	t (df)	*p*
*Fixed effect*	*b*			*Fixed effect*	*b*		
Intercept	6.98 [6.61, 7.35]			Intercept	8.08 [7.49, 8.66]		
Motivation to watch	−0.21 [−0.30, −0.13]	5.36 (51.28)	< .001	Motivation to watch	−0.12 [−0.21, −0.03]	2.77 (39.13)	.009
Motivation to socialize	−0.08 [−0.20, 0.05]	1.24 (43.99)	.221				
Motivation to interact	0.04 [−0.08, 0.16]	0.63 (88.84)	.528				
*Random effects*	*SD*			*Random effects*	*SD*		
Participant				Participant			
Intercept	2.88			Intercept	3.00		
Motivation to watch	0.03			Motivation to watch	.09		
Motivation to socialize	0.18			Residual	1.52		
Motivation to interact	0.09						
Residual	1.53						

Behavior: Socializing with children			
Term	Estimate [95% CI]	t (df)	*p*
*Fixed effect*	*b*		
Intercept	0.12 [−0.02, 0.27]		
Motivation to watch	0.01 [−0.03, 0.05]	0.63 (351.63)	.529
Motivation to socialize	0.05 [−0.05, 0.16]	1.04 (114.60)	.303
Motivation to interact	−0.02 [−0.10, 0.06]	0.50 (534.58)	.619
*Random effects*	*SD*		
Participant			
Intercept	.34		
Motivation to watch	.04		
Motivation to socialize	.35		
Motivation to interact	.05		
Residual	1.47		

Behavior: Sexually interacting with children			
Term	Estimate [95% CI]	t (df)	*p*
*Fixed effect*	*b*		
Intercept	6.98 [6.61, 7.35]		
Motivation to watch	−0.21 [−0.30, −0.13]	.37 (342.83)	.709
Motivation to socialize	−0.08 [−0.20, 0.05]	.80 (182.96)	.428
Motivation to interact	0.04 [−0.08, 0.16]	.38 (570.93)	.706
*Random effects*	*SD*		
Participant			
Intercept	.11		
Motivation to watch	.01		
Motivation to socialize	.05		
Motivation to interact	.02		
Residual	.29		

*Note.* For fixed effects, estimates are unstandardized regression coefficients. For random effects, the standard deviation of the intercepts and slopes is displayed.

**Figure 2.**
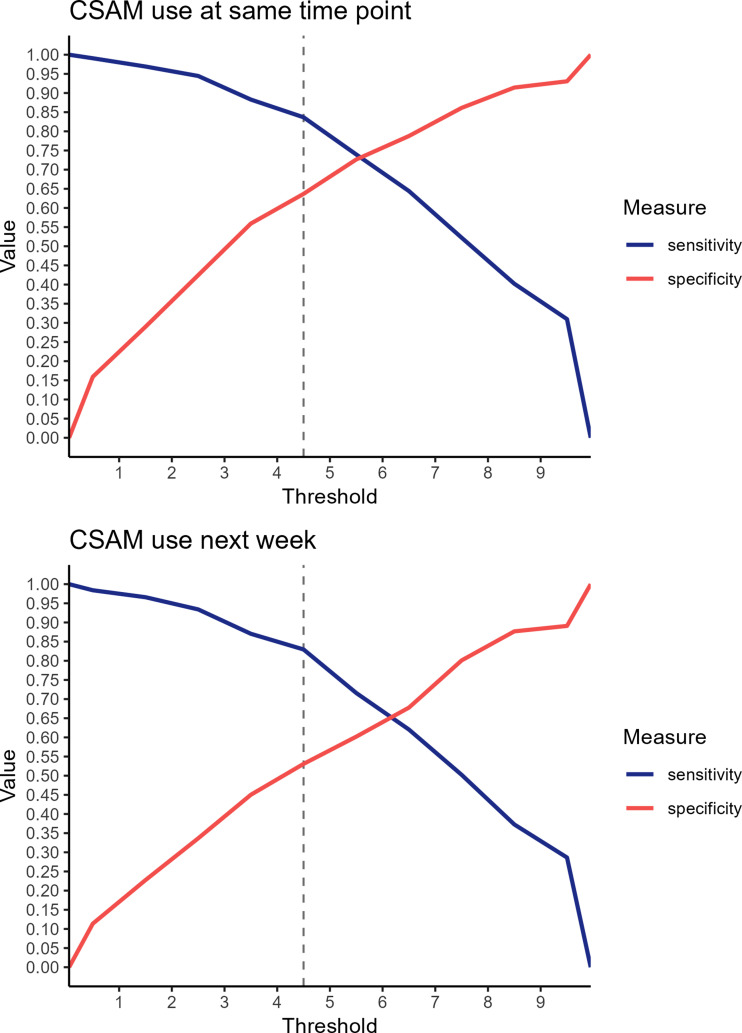
Roc analysis for motivation to watch CSAM predicting CSAM use in prevent it *Note.* Sensitivity (probability of true positives) and specificity (probability of true negatives) are plotted on the *y*-axis, and potential threshold values are plotted on the *x*-axis. A *vertical* dashed line is plotted at the identified cutoff value

The ROC analysis examining the ability of self-reported motivation to watch CSAM to predict contemporaneous use of CSAM (see Figure 2, top panel) and prospective use of CSAM (see Figure 2, bottom panel) yielded similar results. Both analyses indicated an optimal cutoff value of 4.5. For measures at the same time point, this cutoff provided 63.7% specificity and 83.7% sensitivity (AUC = .802, 95% CI [.769, .836]), and for the next week, this cutoff provided 53.1% specificity and 83.0% sensitivity (AUC = .727, 95% CI [.684, .770]).

#### Static Risk

The static risk measure based on the Static-99R* that was used in the Prevent It trial did not significantly predict CSAM use, *b* = −0.72 [−1.68, 0.23], *t* (56.20) = 1.52, *p* = .134, COPINE severity, *b* = 0.16 [−0.21, 0.52], *t* (50.42) = 0.87, *p* = .387, the average age of youngest child, *b* = −0.17 [−0.67, 0.33], *t* (48.24) = 0.68, *p* = .502, socializing with children, *b* = 0.07 [−0.08, 0.22], *t* (80.52) = 0.89, *p* = .375, or sexually interacting with children, *b* = 0.01 [−0.01, 0.04], *t* (64.48) = 1.00, *p* = .323.

## Discussion

Understanding and effectively addressing the risks associated with individuals engaging in harmful sexual behaviors towards children is a complex challenge in the health care and criminal justice systems. This study aimed to investigate the predictive ability of the SChiMRA+, particularly for those involved in CSAM offenses. When assessing SChiMRA and SChiMRA+, the results showed that self-reported motivation to watch CSAM predicted CSAM use within the same week and, for SChiMRA+, also the following week. Given these significant correlations and considering sensitivity and specificity, we recommend a cutoff score of 4.5 for both SChiMRA and SChiMRA+ in similar clinical populations: help-seeking, non-justice-involved online and face-to-face contexts in which archival data are unavailable. The results suggest that self-reports that do not involve direct admission of behavior can serve as useful predictors of subsequent disclosure of behavior under semi-anonymous conditions. Therefore, the cut-off can serve as an indicator of potential risk for engaging in CSAM-related behaviors. However, sensitivity and specificity are influenced by sample characteristics, follow-up time, and outcome base rates, which can vary across studies, populations, and time frames, potentially affecting the generalizability of this cutoff (Helmus & Babchishin, 2017). The evaluation of the SChiMRA+ in the context of clinical trials, along with comparisons to the widely used Static-99R* tool, provides a nuanced understanding of its predictive capabilities.

By incorporating self-reported data, SChiMRA + provides a more comprehensive view of individuals’ sexual behaviors and urges than using register data alone. The assessment has high versatility, successfully applied to both anonymous internet-based populations and in-person participants. The evaluation of Part A (sexual motivation) and Part B (sexual behaviors) allowed for a nuanced understanding of both the desire and the behavioral aspects, filling gaps in traditional risk assessments (Eke et al., 2019). However, it is important to acknowledge that the predictive validity of SChiMRA + has not been independently validated beyond the current study. In the online help-seeking group of CSAM users, self-reported desire to watch CSAM also correlated with watching a higher COPINE severity and younger age of the child in CSAM in that and the following week. This suggests heightened risk during periods of heightened urges. Notably, there were more statistically significant correlations to CSAM severity in the Prevent It online help-seeking group compared to participants in the Priotab medical trial. A plausible explanation is that the Prevent it group was recruited through darknet forums dedicated to CSAM, likely reflecting a population with more established CSAM-related behaviors and easier access to severe material. Participants in this group might therefore be more likely to to use CSAM with higher COPINE severity and younger children when experiencing heightened urges, compared to participants in the other group.

There has been skepticism about the ability to obtain accurate and reliable self-reports on sensitive information, such as taboo thoughts and criminal behaviors (Tourangeau & Yan, 2007). However, empirical evidence suggests that well-designed self-report instruments can be valuable in forensic, correctional, and psychiatric settings (Kroner et al., 2020; Rodrigues et al., 2016; Walters, 2006). Participants in the anonymous online sample rated their level of truthfulness each week, consistently reporting high levels across each measurement point and treatment condition. At the same time, they also consistently disclosed taboo urges and criminal behaviors. Self-rated truthfulness is not direct evidence of actual truthful responding, however these consistent patterns provide indications of participants’ willingness to self-report sensitive, and even incriminating, information. This could possibly be attributed to the anonymity provided in the online setting. However, even in the pseudonymized Priotab medical trial, where there was face to face contact, SChiMRA was still able to detect that the desire to use CSAM predicted self-reported CSAM use. This suggests that SChiMRA effectively captures both the motivation and actual engagement in CSAM use without requiring full anonymity, supporting the generalizability of the findings to pseudonymized in-person clinical settings.

Studies on the characteristics of individuals with child sexual abuse offenses reveal three differentiable groups: those that access CSAM, those who commit contact offenses, and those that commit both contact and CSAM offenses (Babchishin et al., 2018; Babchishin et al., 2015; Hanson & Bussière, 1998; Hanson & Morton-Bourgon, 2005; Ly et al., 2016, 2018; Seto, 2013; Seto et al., 2011). Due to these distinguishable groups, there is a need to use specific or adapted risk assessment tools to accurately predict different types of sexual offending behaviors for different target groups. Studies examining the predictive validity of Static-99R and CPORT have highlighted their limitations in assessing CSAM-related behaviors, particularly among non-convicted populations (Eke et al., 2019; Helmus et al., 2024). When assessing the Static-99R*’s predictive ability on the largely non-convicted populations in Priotab and Prevent It we found that it was not effective in predicting CSAM use in either population; there was not strong enough data to assess a correlation between assessed risk and sexually offending. This provides support for the evidence suggesting that Static-99R* is not suitable for non-convicted populations. Because of these limitations, it is possible that CSAM use among those with contact offenses have been underestimated in the past, partially due to the lack of specific questionnaires. Given that many studies are dated and access to CSAM has increased, current estimates of CSAM use and associated risk factors may be inaccurate; updated research is warranted.

Our study extends the existing literature by demonstrating the efficacy of SChiMRA+ in capturing nuanced aspects of risk among help-seeking individuals with CSAM-related concerns. This emphasizes the importance of tailored risk assessment tools for specific subgroups, such as those experiencing urges to access CSAM or those with undetected CSAM and/or contact offenses. Furthermore, by identifying significant correlations between anonymous self-reported urges and actual CSAM use, our findings underscore the value of incorporating self-reported subjective data into risk assessments in a way that encourages truthful reporting (Tourangeau & Yan, 2007).

As shown in Figure 1, the majority of participants in Prevent It at baseline seemed to have high risk for CSAM use, but lower risk for socializing or interacting sexually with children. SChiMRA+ was able to capture each item’s predictive ability independent from each other in the Prevent It sample, showing that wanting to use CSAM predicted CSAM use, wanting to socialize predicted socializing, and wanting to sexually interact predicted sexually interacting. There were no cross predictions, such as wanting to use CSAM predicting sexually interacting, which could reflect the current literature on group differences showing that those in the CSAM group may be at lower risk of crossing over to contact offending (Babchishin et al., 2018).

In conclusion, our findings show that SChiMRA + can be effective in identifying individuals at increased risk of CSAM use (using a cut-off value of 4.5). In a clinical context, this score allows therapists to be more aware of changes in urges of patients who view or have viewed CSAM, so they can act more preventatively and increase support when needed. Additionally, the cutoff score on urges can be used as a proxy measure for actual CSAM use, which may be valuable in many jurisdictions where strict reporting obligations make it impossible to inquire about actual behaviors. It may also be useful in non-anonymous clinical settings, where patients might hesitate to disclose criminal behaviors but feel safer reporting urges. However, given that all prior studies using the SChiMRA + questionnaire have been based on the two samples included in the current study, and its predictive validity has not yet been independently examined, caution should be exercised when considering its clinical utility. With this we advocate for the use of SChiMRA+, a time-sensitive tool, particularly in measuring the risk of CSAM use among help-seeking, non-justice-involved online and face-to-face contexts in which archival data are unavailable, which are common scenarios while working with helplines and clinical trials.

## Limitations

While this study provides valuable insights into risk assessment for individuals engaged in harmful sexual behaviors, there are several limitations that should be considered when interpreting the findings. First, the study relies on data from specific clinical trials, namely Priotab and Prevent It, and while these capture different types of at-risk persons from all world regions, they may not be fully representative of the broader population of individuals involved in sexual offenses against children. Specifically, all participants in the trials were help-seeking, referred by support communities, helplines, and therapists, or recruited from darknet forums. Second, the use of self-reported data, introduces the possibility of response bias. Participants may underreport or overreport certain behaviors due to factors such as social desirability or fear of consequences, particularly in the context of SChiMRA + that measures stigmatized and criminalized behavior. Priotab was a pseudonymized trial and Prevent It was a fully anonymous trial, which we believe increased the accuracy and willingness to share sensitive and incriminating information. Both samples did, in fact, consistently report sensitive information, and Prevent It participants in general rated their responses as highly truthful. It is possible that this willingness to report truthfully, particularly regarding criminal behaviors, is lower in non-anonymous samples. While the patients had reduced incentives to provide false responses due to the (semi) anonymous and non-punitive context, we recognize that this cannot fully eliminate the possibility of socially desirable responding. Regarding the data and analyses, the following factors need to be considered. The study primarily analyzes the predictive ability of SChiMRA+ within the same week, offering a cross-sectional perspective. This approach limits our ability to assess long-term predictive validity. Future research incorporating longitudinal data could provide a more robust understanding of how motivation relates to subsequent behaviors over time. Due to the assessment schedule in the Priotab trial, our study was unable to assess the predictive ability of “wanting” ratings from one week to subsequent “behavioral” items in the following week. This limits the ability to analyze the relationship between motivation and behaviors over time, within the Priotab sample.

Lastly, the analysis of Static-99R*’s predictive ability is hindered by incomplete data, particularly in the Prevent It trial where three questions were omitted. This limitation compromises the tool’s full applicability in assessing CSAM reoffending risk. Additionally, we did not administer the CPORT in Priotab or Prevent It, largely because (a) both trials pre-dated recent applications of CPORT in non-justice-involved, help-seeking samples and (b) several CPORT items rely on official criminal-justice data that cannot be reliably captured in fully anonymous designs without increasing legal-risk concerns for participants. Nonetheless, emerging work suggests CPORT may have utility beyond justice-involved cohorts (Helmus et al., 2024), which may make it a more appropriate comparison than Static-99R*. Given our focus on week-to-week prediction of motivation and behavior versus CPORT’s primarily static, baseline recidivism classification, these tools answer complementary questions.

## Conclusion and Future Direction

In conclusion, this study offers valuable contributions to the ongoing discussion of risk assessment for non-convicted individuals involved in CSAM offenses by evaluating the predictive ability of SChiMRA+. By establishing an empirically based cut-off value of 4.5, we now have a tool for identifying increased risk as well as probable ongoing CSAM use among self-identified, anonymous or pseudo-anonymous help-seekers. While this tool has only been shown to be applicable in clinical trials, it could reasonably also be used in a therapeutic context as a time sensitive measure of risk.

Future studies should validate SChiMRA+ with information from the crime registry or other objective data sources and test its validity with identified face-to-face clients. Moving forward, a more tailored and nuanced approach to risk assessment, considering individual characteristics and behaviors, can hopefully enhance preventive strategies and promote effective interventions for persons at risk of sexually offending against children. Future directions can also focus on evaluating SChiMRA + as a risk assessment for grooming or contact offending.

Validating SChiMRA + requires testing the cut-off value of 4.5, found by assessing its sensitivity and specificity. These metrics depend on sample characteristics, follow-up time, and base rates, making cut off scores variable across contexts. Future research should assess SChiMRA + across diverse settings and longitudinal studies to refine stable and reliable threshold determinations.

Lastly, in the context of cultural adaptations, the behavioral items of SChiMRA + can be omitted during clinical trials in countries where national reporting laws impose stricter regulations. This modification reflects the sensitivity to legal and cultural variations, emphasizing the importance of aligning the tool with the prevailing norms and legal frameworks of different countries. Future studies could delve deeper into the implications of these adaptations, exploring the impact on the tool’s effectiveness and cultural relevance. Research could also assess the validity and reliability of the adapted versions to ensure that the modifications do not compromise the tool’s ability to accurately capture risk-related information in diverse cultural contexts.

## Other Declarations

Role of funding: This study had strategic and financial support from the World Childhood Foundation and was also funded by the Swedish Society of Medicine, the Söderström Königska Foundation, the Fredrik and Ingrid Thuring Foundation, the OAK Foundation, and the Centre for Psychiatry Research, Department of Clinical Neuroscience, Karolinska Institutet. Finally, the author AM received a scholarship from the Krica Foundation. The funding sources were not involved in the data collection, analysis or preparation of this manuscript.

## Supplemental Material

Supplemental Material - Predictive Ability of the Sexual Child Molestation Risk Assessment (SChiMRA+) Supplemental Material for Predictive Ability of the Sexual Child Molestation Risk Assessment (SChiMRA+) by Allison McMahan, Timothy J. Luke, Gerhard Andersson, Christoffer Rahm and Malin Joleby in Sexual Abuse

Supplemental Material - Predictive Ability of the Sexual Child Molestation Risk Assessment (SChiMRA+) Supplemental Material for Predictive Ability of the Sexual Child Molestation Risk Assessment (SChiMRA+) by Allison McMahan, Timothy J. Luke, Gerhard Andersson, Christoffer Rahm and Malin Joleby in Sexual Abuse

## Data Availability

Data and material not available on request due to ethics approval. Code used to analyze the data available upon request.*
