# Supplementary material for: Predictive Ability of the Sexual Child Molestation Risk Assessment (SChiMRA+)
Source: Sex Abuse. 2026 Jan 12;38(4):447–73. doi: 10.1177/10790632261415813 (PMC13083816; doi:10.1177/10790632261415813)
Supplement: Supplemental Material - Predictive Ability of the Sexual Child Molestation Risk Assessment (SChiMRA+) [file sj-pdf-1-sax-10.1177_10790632261415813.pdf]

SChiMRA

Part A

How likely is it that you would do any of the following if there was an easy way to do it without being detected? Mark an X on the line under each question

1) Watch

Watch child sexual abuse material, pictures or films, or discreetly observe children/youths for sexual arousal?

Very likely Not at all

____

2) Socialize

Socialize/talk to/chat online/call/text/send letters to children/youths for sexual arousal, or in the hopes it may later lead to something more?

Very likely Not at all

____

3) Interact sexually

Have physical contact with a child/youth for pleasure or sexual enjoyment, or encourage the child/youth into touching you, or stage other types of more direct sexual/sensual situations remotely (for example through webcam)?

Very likely Not at all

____

©2020 American Medical Association. All rights reserved.

SChiMRA

Part B

Think about the last seven days. How often have you engaged in some of the following:

1) Watched

Watched child sexual abuse material, pictures or films, or discreetly observed children/youths for sexual arousal?

Not at all	A few days	More than half the days	Nearly every day
☐	☐	☐	☐

Comment: ____

2) Socialized

Socialized/talked to/chatted online/texted/sent letters to children/youths for sexual arousal, or in the hopes it may later lead to something more?

Not at all	A few days	More than half the days	Nearly every day
☐	☐	☐	☐

Comment: ____

3) Interacted sexually

Have physical contact with a child/youth for pleasure or sexual enjoyment, or encourage the child/youth into touching you, or stage other types of more direct sexual/sensual situations remotely (for example through webcam)?

Not at all	A few days	More than half the days	Nearly every day
☐	☐	☐	☐

Comment: ____

© 2020 Landgren V et al. *JAMA Psychiatry.*
