# Supplementary material for: Predictive Ability of the Sexual Child Molestation Risk Assessment (SChiMRA+)
Source: Sex Abuse. 2026 Jan 12;38(4):447–73. doi: 10.1177/10790632261415813 (PMC13083816; doi:10.1177/10790632261415813)
Supplement: Supplemental Material - Predictive Ability of the Sexual Child Molestation Risk Assessment (SChiMRA+) [file sj-pdf-2-sax-10.1177_10790632261415813.pdf]

Sexual Child Molestation Risk Assessment (SChiMRA+)

Rahm Research Group
Center for Psychiatry Research
Karolinska Institutet

The SChiMRA+ is a self-assessment designed for help seeking individuals with sexual behaviors towards children, and is available in English, Swedish, German, Finnish, Portuguese, Spanish, and Czechian.

SChiMRA+ has been shown to be suitable for clinical trials and sensitive to change both for iCBT and Testosterone lowering medication treatments. This sensitivity is decreased for placebo (psychological or medical) groups.

Part A asks questions related to self-rated risk, or 'wanting' to engage with children, while Part B covers past week behaviors, including time spent using CSAM (B1), socializing (B2) or physically interacting with children for sexual arousal (B3).

An individual is considered high risk for committing CSAE if Part A item 2 OR item 3 is greater that 3, OR Part B item 2 OR item 3 is greater than 0.

Please consult a lawyer regarding reporting obligations in your country in order to inform patients before filling in the questionnaire. Be aware, strict reporting obligations can bias the reliability of the answers since they are self-reported.

Part A

Answer on a scale from 0-10, where:
0 = Not at all
10 = Very likely
Question left unanswered equals x

How likely is it that you would do any of the following if there was an easy way to do it without being detected?

1) *Watch*
   Watch child sexual abuse material, pictures or films, or discreetly observe children/youths for sexual arousal? 0-10

2) *Socialize*
   Socialize/talk to/chat online/call/text/send letters to children/youths for sexual arousal, or in the hopes it may later lead to something more? 0-10

3) *Interact sexually*
   Have physical contact with a child/youth for pleasure or sexual enjoyment, or encourage the child/youth into touching you, or stage other types of more direct sexual/sensual situations remotely (for example through webcam)? 0-10

Part B

Answers from 0-3, with the options:
0 = Not at all,
1 = A few days (1-3 days),
2 = more than half the days,
3 = nearly every day
Question left unanswered equals x

Think about the last seven days. How often have you engaged in some of the following:

1) *Watched*
   Watched child sexual abuse material, pictures or films, or discreetly observed children/youths for sexual arousal? 0-3

   If answered with 1-3 points:

   1. How many days in the last week? (1-7)
   2. How much time was spent during each day? If you do not know, put an x in the box for that day.

Day	Time (hours and minutes)
1	00:00
2	
3	
etc	

   3. Which would be the highest level of the COPINE scale you watched during each day? If you do not know, put an x in the box for that day.

Day	COPINE scale
1	
2	
3	
etc	

   4. Estimate the youngest child you watched each day. If you do not know put an x in the box for that day.

Day	Age of the youngest child
1	
2	
3	
etc	

2) *Socialized*
   Socialized/talked to/chatted online/texted/sent letters to children/youths for sexual arousal, or in the hopes it may later lead to something more? 0-3

   If answered with 1-3 points:

   1. How many days in the last week? (1-7)

2. How much time was spent during each day? If you do not know, put an x in the box for that day.

Day	Time (hours and minutes)
1	
2	
3	
etc	

3. Estimate the youngest child you socialized with each day. If you do not know put an x in the box for that day.

Day	Age of the youngest child
1	
2	
3	
etc	

3) *Interacted sexually*
Have physical contact with a child/youth for pleasure or sexual enjoyment, or encourage the child/youth into touching you, or stage other types of more direct sexual/sensual situations remotely (for example through a webcam)? 0-3

If answered with 1-3 points:

1. How many days in the last week? (1-7)
2. How much time was spent during each day? If you do not know, put an x in the box for that day.

Day	Time (hours and minutes)
1	
2	
3	
etc	

3. Estimate the youngest child you interacted sexually with each day. If you do not know put an x in the box for that day.

Day	Age of the youngest child
1	
2	
3	
etc	

4) Other behaviors related to your sexual interest in children
Searching for material, interacting (e.g chatting) with other people about children, categorizing material or fantasizing about children? 0-3

If answered with 1-3 points:

1. How many days in the last week?
2. How much time was spent during each day? If you do not know, put an x in the box for that day.

Day	What kind of behavior	Time (hours and minutes)
1		
2		
3		
etc		

3. Estimate the youngest child you exhibited behaviors towards/arounds each day. If you do not know put an x in the box for that day.

Day	Age of the youngest child
1	
2	
3	
etc	

COPINE

When watching material depicting children to gain sexual arousal, what kind of material do you normally use? Mark one or more of the following options:
Question left unanswered equals x

1. Indicative (equals 0)
Non-erotic and non-sexualized pictures showing children in their underwear, swimming costumes from either commercial sources or family albums. Pictures of children playing in normal settings, in witch the context or organization of pictures by the collector indicates inappropriateness.

2. Nudist (equals 1)
pictures of naked or semi-naked children in appropriate nudist settings, and from legitimate sources

3. Ero (equals 2)
Surreptitiously taken photographs of children in play areas or other safe environments showing either underwear or varying degrees of nakedness

4. Posing (equals 3)
Deliberately posed pictures of fully clothed, partially clothed or naked (where the amount, context and organization suggests sexual interest)

5. Erotic Posing (equals 4)
Deliberately posed pictures of fully, partially clothed or naked children in sexualized or provocative poses

6. Explicit Erotic Posing (equals 5)
Pictures emphasizing genital areas, where the child is either naked, partially clothed or fully clothed

7. Explicit Sexual Activity (equals 6)

Pictures that depict touching, mutual and self-masturbation, oral sex and intercourse by a child, not involving an adult

8. Assault (equals 7)
Pictures of children being subject to a sexual assault, involving digital touching, involving an adult

9 Gross Assault (equals 8)
Grossly obscene pictures of sexual assault, involving penetrative sex, masturbation or oral sex, involving an adult

10. Sadistic/Bestiality (equals 9)
a) Pictures showing a child being tied, bound, beaten, whipped or otherwise subject to something that implies pain
b) Pictures where an animal is involved in some form of sexual behavior with a child
